# Increasing Rates of Obesity among HIV-Infected Persons during the HIV Epidemic

**DOI:** 10.1371/journal.pone.0010106

**Published:** 2010-04-09

**Authors:** Nancy Crum-Cianflone, Mollie Poehlman Roediger, Lynn Eberly, Maryam Headd, Vincent Marconi, Anuradha Ganesan, Amy Weintrob, R. Vincent Barthel, Susan Fraser, Brian K. Agan, the Infectious Disease Clinical Research Program HIV Working Group

**Affiliations:** 1 Infectious Disease Clinical Research Program, Uniformed Services University of the Health Sciences, Bethesda, Maryland, United States of America; 2 Infectious Disease Clinic, Naval Medical Center San Diego, San Diego, California, United States of America; 3 Graduate School of Public Health, San Diego State University, San Diego, California, United States of America; 4 Division of Biostatistics, University of Minnesota, Minneapolis, Minnesota, United States of America; 5 Infectious Disease Clinic, San Antonio Military Medical Center, San Antonio, Texas, United States of America; 6 Infectious Disease Clinic, National Naval Medical Center, Bethesda, Maryland, United States of America; 7 Infectious Disease Clinic, Walter Reed Army Medical Center, Washington, D.C., United States of America; 8 Infectious Disease Clinic, Naval Medical Center Portsmouth, Portsmouth, Virginia, United States of America; 9 Infectious Disease Clinic, Tripler Medical Center, Honolulu, Hawaii, United States of America; University of Toronto, Canada

## Abstract

**Background:**

The prevalence and factors associated with overweight/obesity among human immunodeficiency virus (HIV)-infected persons are unknown.

**Methods:**

We evaluated prospective data from a U.S. Military HIV Natural History Study (1985–2004) consisting of early diagnosed patients. Statistics included multivariate linear regression and longitudinal linear mixed effects models.

**Results:**

Of 1682 patients, 2% were underweight, 37% were overweight, and 9% were obese at HIV diagnosis. Multivariate predictors of a higher body mass index (BMI) at diagnosis included more recent year of HIV diagnosis, older age, African American race, and earlier HIV stage (all p<0.05). The majority of patients (62%) gained weight during HIV infection. Multivariate factors associated with a greater increase in BMI during HIV infection included more recent year of diagnosis, lower BMI at diagnosis, higher CD4 count, lower HIV RNA level, lack of AIDS diagnosis, and longer HIV duration (all p<0.05). Nucleoside agents were associated with less weight gain; other drug classes had no significant impact on weight change in the HAART era.

**Conclusions:**

HIV-infected patients are increasingly overweight/obese at diagnosis and during HIV infection. Weight gain appears to reflect improved health status and mirror trends in the general population. Weight management programs may be important components of HIV care.

## Introduction

Obesity rates among the general population have steadily risen [1]; however data on weight trends among human immunodeficiency virus (HIV)-infected persons are sparse. Most prior studies have had significant limitations including their cross-sectional study designs, lack of longitudinal weight measurements, evaluation of only a single clinic setting, or their focus on wasting alone [2]–[5]. However, it is likely that as HIV-infected patients are living longer [6], [7] and experiencing lower rates of acquired immunodeficiency syndrome (AIDS)-related wasting syndrome [8]–[11] due to the beneficial effects of highly active antiretroviral therapy (HAART), they may become overweight or obese at a rate similar to that of the general U.S. population and suffer from medical comorbidities related to excess weight.

No study to date has provided data on the weight trends among HIV-infected patients over the course of the HIV epidemic. In addition, there is a paucity of longitudinal data on the factors associated with weight changes during the course of an individual's HIV infection. Therefore, we evaluated prospectively collected data to assess weight trends during the epidemic and examined factors associated with weight changes among individual patients during HIV infection.

## Methods

We examined prospectively collected data as part of the U.S. Military Natural History Study, a multicenter observational study, which enrolled 4586 HIV-infected persons from 1985–2004 at seven U.S. geographic locations. From the total study cohort, all participants were included in the current analyses except if they were less than 18 years of age, they did not have a baseline height measurement recorded, or they did not have a baseline weight measurement within one year of HIV diagnosis. Participants were military beneficiaries (active duty, retirees, and dependents); active duty members are HIV negative upon service entry and undergo mandatory testing every 1–5 years. Participants are evaluated on a biannual basis and weight measurements, medical conditions, and medications are collected utilizing standardized collection procedures.

Data collected at HIV diagnosis (baseline) included: weight and height; demographics (age, gender, self-reported race/ethnicity); military duty status; Walter Reed stage designated 1–6 for ascending degrees of disease based on cluster of differentiation 4 (CD4) counts, opportunistic infections, lymphadenopathy, and delayed-type hypersensitivity [12]; CD4 counts; HIV ribonucleic acid (RNA) levels (including a category for ‘missing’ as viral loads were not routinely collected until 1996); and medical history. Data collected at each follow-up visit included weight, CD4 counts, HIV RNA levels recorded as copies/milliliter (mL) by Roche, Amplicor assay, antiretroviral therapy prescription dates, and updated medical history. Weight measurements were obtained at the initial evaluation and at each six-month visit; patients were weighed on calibrated scales at each site, and measurements were taken with patients' clothes on. The study period was *a priori* divided into two pre-HAART periods (1985–1990 and 1991–1995) and two HAART periods (1996–2000 and 2001–2004); since there were no significant differences in outcomes between the two HAART periods, these were combined into 1996–2004 for some analyses. Body mass index (BMI) at baseline and each semiannual visit was categorized with <18.5 kilogram per meter squared (kg/m^2^) as underweight, 18.5–24.9 kg/m^2^ as normal weight, 25–29.9 kg/m^2^ as overweight, and ≥30 kg/m^2^ as obese [13], [14]. All participants were evaluated for baseline BMI (n = 1682) and participants with ≥1 year of follow-up were also evaluated longitudinally from time of HIV diagnosis to last study visit (n = 1255); the mean number of weight measurements per participant was 9 with a standard deviation (SD) of 6. Incident cases of becoming underweight, overweight, or obese were confirmed by two consecutive measurements during follow-up. All participants included in this report were diagnosed with HIV infection from 1985 to 2004. The last weight measurement was obtained on April 23, 2007.

Our study was approved by the central governing Institutional Review Board which is located at Wilford Hall Medical Center, Lackland Air Force Base, San Antonio, Texas. The study was conducted according to the principles expressed in the Declaration of Helsinki. All study participants provided written informed consent.

Statistical analyses utilized Fisher's exact tests for baseline BMI group comparisons for categorical variables and Kruskal-Wallis tests for continuous measurements. Predictors of BMI at baseline were tested in univariate and multivariate linear regression models. Least squares means (adjusted to the sample's marginal frequencies) were computed for predicted BMI by baseline subgroups. The multivariate model was adjusted for age, gender, race/ethnicity, year of HIV diagnosis, baseline CD4 count and HIV RNA level, military duty status, and Walter Reed stage. The multivariate model was repeated to examine the subgroup of documented seroconverters.

Comparisons across BMI categories at the time of last visit for the incidence (between baseline and last visit) of hypertension, hyperlipidemia, diabetes, or heart disease utilized Fisher's exact tests. Patients diagnosed with the condition prior to HIV diagnosis were excluded from these specific analyses.

Change in BMI from baseline was computed at each follow-up visit among participants with a BMI measurement at HIV diagnosis and at least one weight measurement ≥1 year after diagnosis. Using longitudinal linear mixed effects models with random intercept and random slope across years of follow-up for each participant, regressions for change in BMI were fitted using all follow-up data. Factors of interest included age, gender, race/ethnicity, baseline BMI, year of HIV diagnosis, baseline Walter Reed stage, and time-updated variables for active duty status, CD4 count, HIV RNA level, an AIDS-defining illness, and cumulative time receiving antiretroviral therapy (ART). All time-updated covariates represented the most recently observed value at or prior to each BMI measurement. The multivariate model was adjusted for all factors of interest, as well as for follow-up time. Least square means (adjusted to the sample's marginal frequencies) were computed for predicted change in BMI over follow-up for each categorical factor. Additional multivariate models were performed examining: 1) the subgroup of participants diagnosed in the HAART era (since 1 Jan 1996), 2) this subgroup before initiation of ART, and 3) this subgroup after initiation of ART. Missing BMI values were not imputed. Results were considered statistically significant for P-values<0.05. All analyses were conducted using SAS (version 9.1, SAS Institute, Cary, NC).

## Results

### Baseline Characteristics and Weight Measurements at HIV Diagnosis

From the total study cohort, participants were excluded from the current analyses if they were less than 18 years of age (n = 0), they did not have a baseline height measurement recorded (n = 419), or they did not have a baseline weight measurement within one year of HIV diagnosis (n = 2870); the later exclusion was mainly a result of participant enrollment into the study cohort >1 year after diagnosis. In total, 1682 participants met inclusion criteria; this group was older (31 vs. 30 years), more likely male (93% vs. 90%), Caucasian (51% vs. 45%), active duty (83% vs. 63%), and had earlier stage infection (66% vs. 50%) than those excluded from the analysis (p<0.05). Of the 1682 participants, 1367 (81%) were documented HIV seroconverters with a median seroconversion window of 16.9 months (IQR: 10.3–29.2).

Table 1 shows the baseline characteristics of the 1682 participants at HIV diagnosis. Across the entire study period (1985–2004), at the time of HIV diagnosis, 31 (2%) were underweight, 871 (52%) were normal weight, 623 (37%) were overweight, and 157 (9%) were obese. The mean BMI at diagnosis was 25.0 (SD 3.8, range from 12.3 to 45.7). Among men (n = 1556), 1% were underweight, 52% were normal weight, 38% were overweight, and 9% were obese. Comparatively, women (n = 126) were more likely to be underweight (9%, p<0.001) and obese (17%, p = 0.006), but less likely to be overweight (24%, p = 0.002). Overall, the mean BMI of men and women was similar (25.0 vs. 25.4, p = 0.42).

**Table 1:** Baseline Characteristics by Weight Category.

	Weight Category				
	Underweight	Normal	Overweight	Obese	*P* *
**No.**	31	871	623	157	
**Age**					
Mean ± SD	34.0±13.8	29.6±8.8	31.0±8.1	32.8±9.0	<.001
**Gender**					<.001
Male	20 (1.3%)	807 (51.9%)	593 (38.1%)	136 (8.7%)	
Female	11 (8.7%)	64 (50.8%)	30 (23.8%)	21 (16.7%)	
**Race/Ethnicity**					<.001
White/non-Hispanic/Other	22 (2.6%)	471 (55.3%)	296 (34.8%)	62 (7.3%)	
African American	6 (0.8%)	348 (49.0%)	274 (38.6%)	82 (11.5%)	
Hispanic/Latino	3 (2.5%)	52 (43.0%)	53 (43.8%)	13 (10.7%)	
**Duty Status**					<.001
Active	13 (0.9%)	716 (51.3%)	543 (38.9%)	123 (8.8%)	
Retired	6 (3.9%)	87 (57.2%)	47 (30.9%)	12 (7.9%)	
Dependent	12 (8.9%)	68 (50.4%)	33 (24.4%)	22 (16.3%)	
**CD4 count (cells/mm^3^)**					<.001
Mean ± SD	360.7±287.4	519.3±263.9	539.2±266.9	487.0±234.5	
Median (IQR)	305.0 (180, 489)	493.0 (347, 650)	507.0 (365, 673)	477.0 (324, 637)	
**HIV RNA (log10 copies/mL)**					0.02
Mean ± SD	4.6±1.0	4.3±0.9	4.2±0.9	4.1±1.0	
Median (IQR)	4.9 (4.5, 5.0)	4.4 (3.8, 4.9)	4.3 (3.6, 4.8)	4.3 (3.4, 4.8)	
**HIV RNA levels (copies/mL)**					0.001
** Missing**	16 (51.6%)	397 (45.6%)	219 (35.2%)	55 (35.0%)	
<1,000	2 (6.5%)	42 (4.8%)	53 (8.5%)	13 (8.3%)	
1000–10000	0 (0.0%)	104 (11.9%)	95 (15.3%)	29 (18.5%)	
10000–100000	9 (29.0%)	248 (28.5%)	192 (30.8%)	48 (30.6%)	
>100000	4 (12.9%)	80 (9.2%)	64 (10.3%)	12 (7.6%)	
**Walter Reed stage**					<.001
Stage 1 or 2	12 (1.0%)	622 (51.1%)	477 (39.2%)	106 (8.7%)	
Stage 3 or 4	5 (1.8%)	135 (49.3%)	96 (35.0%)	38 (13.9%)	
Stage 5 or 6	13 (6.9%)	113 (60.1%)	49 (26.1%)	13 (6.9%)	
**Year of HIV Diagnosis**					<.001
1985–1990	7 (2.3%)	211 (69.4%)	77 (25.3%)	9 (3.0%)	
1991–1995	13 (2.2%)	299 (51.0%)	219 (37.4%)	55 (9.4%)	
1996–2004	11 (1.4%)	361 (45.6%)	327 (41.3%)	93 (11.7%)	

*P-values represent differences in proportions between baseline BMI categories were tested using Fisher's exact tests and differences in medians used Kruskal-Wallis tests.

### Factors Associated with BMI at HIV Diagnosis

In the multivariate analysis, older age (p<0.001), African American (p<0.001) compared to Caucasian race, early vs. late Walter Reed stage (p<0.001), and a more recent year of HIV diagnosis (p<0.001) were associated with a higher BMI at HIV diagnosis (Table 2). We repeated our analyses utilizing only documented HIV seroconverters with similar results (data not shown).

**Table 2:** Factors Associated with BMI at Time of HIV Diagnosis.

	Univariate Models			Multivariate Model		
	Mean ± SE	Est. Diff* ± SE	*P* *	Mean ± SE	Est. Diff* ± SE	*P* *
**Age**						
<30	24.52±0.12	**Referent**		24.48±0.12	**Referent**	
30–39	25.73±0.16	1.21±0.20	<0.001	25.69±0.16	1.21±0.20	<0.001
≥40	25.67±0.26	1.15±0.28	<0.001	26.09±0.28	1.61±0.31	<0.001
**Gender**						
Male	25.01±0.10	−0.34±0.35	0.32	25.01±0.09	−0.54±0.42	0.20
Female	25.35±0.33	**Referent**		25.54±0.40	**Referent**	
**Race/Ethnicity**						
White/non-Hispanic/Other	24.67±0.13	**Referent**		24.70±0.13	**Referent**	
African American	25.41±0.14	0.75±0.19	<0.001	25.41±0.14	0.70±0.19	<0.001
Hispanic/Latino	25.37±0.34	0.70±0.36	0.05	25.41±0.33	0.71±0.35	0.05
**Duty Status**						
Active	25.09±0.10	**Referent**		25.08±0.10	**Referent**	
Retired	24.29±0.30	−0.80±0.32	0.01	24.50±0.33	−0.58±0.35	0.10
Dependent	25.25±0.32	0.16±0.34	0.64	25.36±0.39	0.28±0.41	0.49
**Year of HIV Diagnosis**						
1985–1990	23.55±0.21	**Referent**		23.89±0.24	**Referent**	
1991–1995	25.08±0.15	1.53±0.26	<0.001	25.10±0.17	1.21±0.26	<0.001
1996–2004	25.56±0.13	2.01±0.25	<0.001	25.45±0.17	1.57±0.33	<0.001
**CD4 at HIV Diagnosis**						
<500 cells/mm^3^	24.98±0.13	−0.15±0.18	0.42	24.91±0.14	−0.28±0.21	0.18
≥500 cells/mm^3^	25.12±0.13	**Referent**		25.19±0.14	**Referent**	
**HIV RNA levels (copies/mL)**						
Missing	24.55±0.14	−0.50±0.33	0.13	25.03±0.18	0.22±0.38	0.57
<1000	25.68±0.36	0.63±0.46	0.17	25.34±0.36	0.53±0.46	0.25
1000–10000	25.91±0.25	0.86±0.38	0.03	25.47±0.25	0.66±0.38	0.08
10000–100000	24.15±0.17	0.10±0.34	0.77	24.89±0.18	0.08±0.33	0.81
>100000	25.05±0.29	**Referent**		24.81±0.30	**Referent**	
**Walter Reed stage at HIV Diagnosis**						
Stages 1 & 2	25.11±0.11	**Referent**		25.15±0.11	**Referent**	
Stages 3 & 4	25.47±0.23	0.36±0.25	0.15	25.28±0.24	0.13±0.28	0.64
Stages 5 & 6	23.90±0.27	−1.21±0.29	<0.001	24.05±0.28	−1.10±0.31	<0.001

*P-values indicate whether the mean BMI at baseline for groups within a factor are significantly different from that of the referent. The estimated difference represents the difference in mean BMI at baseline between the group and the referent. For example, the estimated difference of 0.70 (p<0.001) for African American race in the multivariate model indicates that the mean baseline BMI was 0.70 kg/m^2^ higher for African American compared to White participants.

### Changes in Weights among HIV-Infected Persons at HIV Diagnosis over the Epidemic

**Figure 1:**
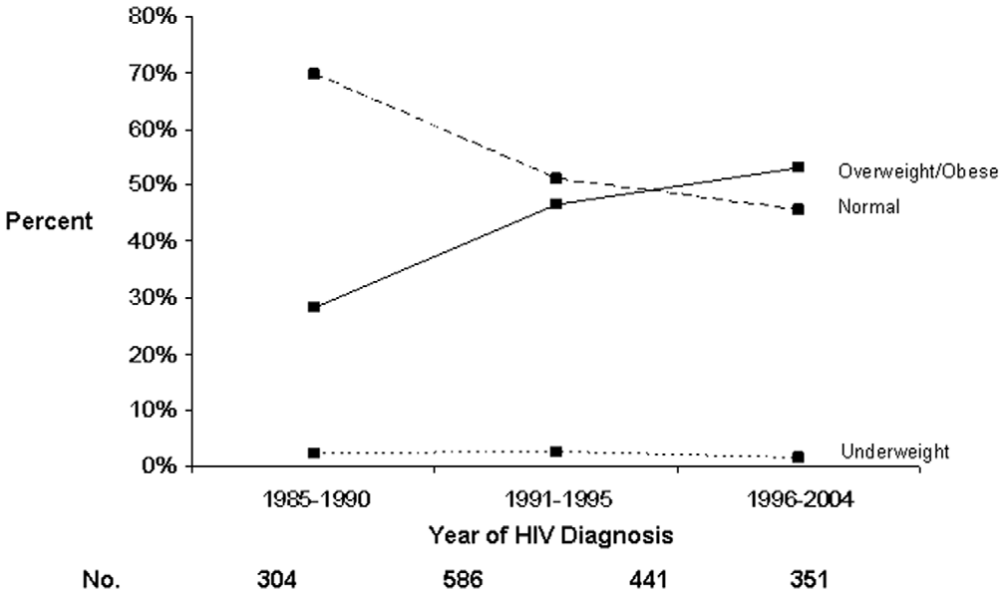
Trends in Weight Categories at HIV Diagnosis during the HIV Epidemic.

**Figure 2:**
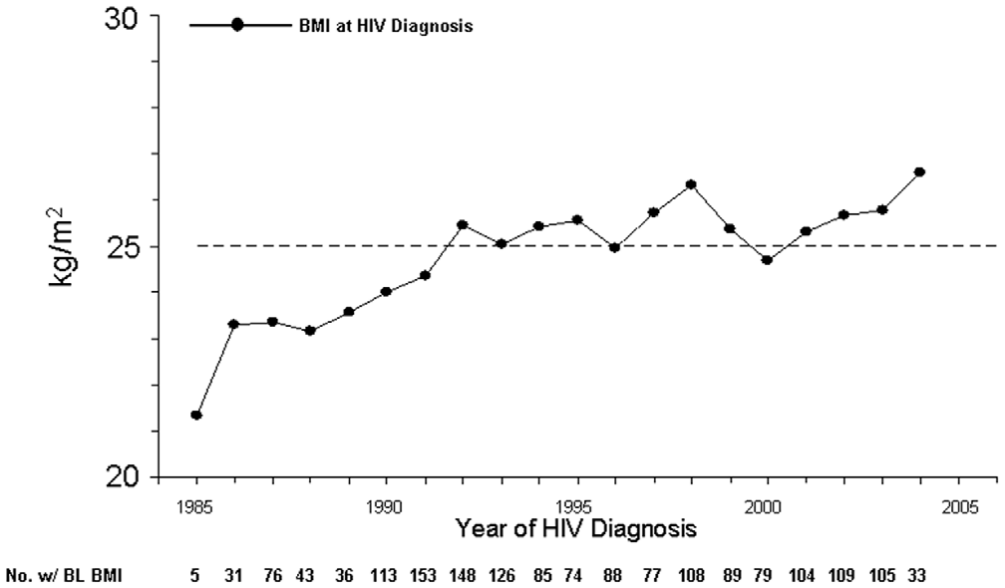
Trends in Mean BMI Measurements at HIV Diagnosis during the HIV Epidemic.

Over the HIV epidemic, the percentage of participants overweight and obese at the time of HIV diagnosis increased (Figure 1) as did the mean BMI (Figure 2). The percentage of participants overweight at HIV diagnosis nearly doubled from 1985–1990 (25%) to 1996–2004 (41%), while obesity increased four-fold (3% vs. 12%, respectively). Being underweight remained uncommon (2%) throughout the 20-year study period. Analyses limited to documented HIV seroconverters produced similar trends.

### BMI and Weight Changes among HIV-infected Persons during the Course of their HIV Infection

Participants (n = 1255) were followed for changes in weight during their HIV infection (mean follow-up 6.2 years, SD 3.9). This group was older (p<0.001) and consisted of fewer women (p = 0.02) than those excluded due to lack of follow-up data. Eighty-one percent of the 1255 received antiretroviral therapy for a mean of 4.4 years (SD 3.3) and HAART was utilized among 65% for a mean of 4.0 years (SD 2.7). The most common antiretroviral class utilized was nucleoside reverse transcriptase inhibitors (NRTIs) taken by 81% of participants; the most common NRTI was zidovudine (71%), followed by lamivudine (67%), stavudine (36%), didanosine (29%), tenofovir (21%), and abacavir (20%). Protease inhibitors (PIs) were used by 47% of participants and included indinavir (24%), nelfinavir (23%), ritonavir (22%), lopinavir (10%). Nucleoside reverse transcriptase inhibitors (NNRTIs) were used by 44%, most commonly efavirenz (39%). The mean CD4 count at initiation of ART was 381 cells/mm^3^ (SD 162) and 384 (SD 200) in the pre- and HAART eras, respectively.

During the course of HIV infection, 61.5% (n = 772) of patients gained weight, 2.9% (n = 36) had no weight change, and 35.6% (n = 447) lost weight. Among those who gained weight, mean increase in BMI and weight was 2.3 kg/m^2^ (SD 2.0) and 7.3 kg (SD 5.9), respectively, from baseline to last visit. The mean BMI and weight gain per year of follow-up was 0.55 kg/m^2^/year and 1.71 kg/year, respectively. Among participants who lost weight, the mean change in BMI and weight was −1.7 kg/m^2^ (SD 1.8) and −5.4 kg (SD 5.5), respectively (mean follow-up time 5.4 years). Of those who lost weight, the mean BMI and weight loss was −0.48 kg/m^2^/year and −1.46 kg/year of follow-up.

Among participants initially underweight at baseline, 24% remained underweight at last visit, while 67% reached a normal weight, 5% became overweight, and 5% became obese. Twenty-six percent who were normal weight at baseline were overweight, and 0.5% was obese at last visit; only 4.5% of those with normal weight progressed to underweight at last visit. The majority of overweight or obese participants at baseline remained in these categories during their HIV infection, with only 1 of 609 becoming underweight.

We also examined the incidence of changes in weight category at anytime during follow-up. The incidence of becoming overweight among participants initially underweight or normal weight (n = 646) was 6.1 per 100 person years (PYs) of follow-up, while the incidence of obesity among overweight participants (n = 487) was 4.3 per 100 PYs. The incidence of becoming underweight among those with an initial BMI>18.5 kg/m^2^ (n = 1234) was only 0.2 per 100 PYs. With an expanded definition to include either the development of a BMI<18.5 kg/m^2^ or a >10% loss of weight from baseline, the incidence was 1.0 per 100 PYs; however, 34% of those who lost >10% were overweight or obese prior to weight loss.

### BMI Measurements at Last Visit and Development of Medical Conditions

At their last visit, 2.7% of HIV-infected patients were underweight, and 56% were overweight (41.5%) or obese (14.9%). Among participants diagnosed during the HAART era (1996–2004, n = 630), 46% were overweight, 16% were obese, and 0.6% were underweight at last visit. Women were more likely than men to be underweight (7% vs. 2%, p = 0.02) and obese (23% vs. 14%, p = 0.05) at last visit; there was a trend that women were less likely overweight (31% vs. 42%, p = 0.06). Mean BMI values at last visit for men and women were the same (26.0 kg/m^2^). The mean BMI at last visit increased over the HIV epidemic in a similar pattern to the BMI values at HIV diagnosis.

Hypertension developed during the course of HIV infection in 0%, 6.2%, 8.6%, and 17.6% of those who were underweight, normal weight, overweight, and obese, respectively (p<0.001). Hyperlipidemia developed in 2.9%, 13.8%, 24.3%, and 28.8% of the four increasing weight categories (p<0.001). Diabetes was diagnosed in 2.9%, 3.0%, 4.8%, and 6.0% of HIV-infected patients, respectively (p = 0.22). Only six participants developed documented heart disease. Similar trends were found after adjusting for age, race, and gender.

### Factors Associated with Longitudinal Weight Trends during HIV Infection

A repeated measures analysis was performed to identify factors associated with changes in BMI from baseline over the course of HIV infection (Table 3, Model 1). All factors had estimated mean changes in BMI that were positive and significantly different from zero (all p-values<0.001) in the multivariate model, except for being obese at baseline (mean change  = 0.18, p = 0.23) and developing an AIDS diagnosis (mean change = 0.03, p = 0.80). Factors associated with a greater increase in BMI in the final multivariate model included a more recent year of HIV diagnosis: participants diagnosed with HIV in 1985–1990 had an average increase in BMI of 0.51 kg/m^2^ compared to increases of 0.62 kg/m^2^ and 0.93 kg/m^2^ for those diagnosed in 1991–1995 and 1996–2004, respectively. Additionally, lower baseline BMI was associated with a greater increase in BMI: participants who were underweight had an average increase in BMI of 2.92 kg/m^2^, while participants who were normal, overweight, or obese had an average increase of 0.89 kg/m^2^, 0.66 kg/m^2^, or 0.18 kg/m^2^, respectively. Other factors associated with greater increases in BMI included a higher time-updated CD4 cell count (p<0.001), a lower time-updated HIV RNA level (all p<0.001 when compared to level >100000 copies/mL), lack of an AIDS diagnosis (p<0.001), and increased time since HIV infection (p<0.001). There were no significant effects of age, gender, race, Walter Reed stage, or active duty status on BMI changes.

**Table 3:** Factors Associated with Changes in BMI.

	Model 1 * : Factors associated with changes in BMI among all participants (N = 1255)			Model 2 * : Factors associated with changes in BMI after initiation of ART among participants diagnosed in the HAART Era (N = 355)		
Factors	Est. Mean Change in BMI	Est. Mean Difference in Change in BMI ± SE	*P* **	Est. Mean Change in BMI	Est. Mean Difference in Change in BMI ± SE	*P* **
**Age**						
<30	0.81‡	**Referent**	**-**	0.70‡	**Referent**	**-**
30–39	0.70‡	−0.11±0.10	0.26	0.39‡	−0.30±0.17	0.07
≥40	0.60‡	−0.21±0.15	0.16	0.78‡	0.08±0.26	0.75
**Gender**						
Male	0.75‡	0.06±0.19	0.75	0.54‡	−0.96±0.40	0.02
Female	0.69‡	**Referent**	**-**	1.50‡	**Referent**	**-**
**Race/Ethnicity**						
White/non-Hispanic/Other	0.71‡	**Referent**	**-**	0.50‡	**Referent**	**-**
African American	0.75‡	0.03±0.10	0.72	0.69‡	0.19±0.17	0.26
Hispanic/Latino	0.96‡	0.24±0.18	0.18	0.53‡	0.02±0.27	0.94
**Active Duty (time updated)**						
No	0.71‡	**Referent**	**-**	0.44‡	**Referent**	**-**
Yes	0.77‡	0.05±0.06	0.35	0.61‡	0.17±0.17	0.32
**Baseline BMI Category**						
Underweight	2.92‡	2.03±0.37	<0.001	1.37‡	0.61±0.70	0.38
Normal	0.89‡	**Referent**	**-**	0.76‡	**Referent**	**-**
Overweight	0.66‡	−0.23±0.10	0.02	0.62‡	−0.14±0.17	0.42
Obese	0.18	−0.71±0.16	<0.001	−0.24	−1.00±0.24	<0.001
**Year of HIV Diagnosis**						
1985–1990	0.51‡	**Referent**	**-**	X	X	X
1991–1995	0.62‡	0.11±0.15	0.46	X	X	X
1996–2004	0.93‡	0.42±0.16	0.009	X	X	X
**Baseline Walter Reed stage**						
Stage 1 or 2	0.75‡	−0.17±0.17	0.32	0.51‡	−0.70±0.32	0.03
Stage 3 or 4	0.65‡	−0.26±0.19	0.18	0.58‡	−0.63±0.33	0.06
Stage 5 or 6	0.91‡	**Referent**	**-**	1.20‡	**Referent**	**-**
**CD4 (time updated)**						
<350 cells/mm^3^	0.59‡	**Referent**	**-**	0.44‡	**Referent**	**-**
≥350 cells/mm^3^	0.79‡	0.20±0.04	<0.001	0.60‡	0.16±0.10	0.10
**HIV RNA levels (time updated copies/mL)**						
Missing	0.71‡	0.37±0.09	<0.001	X	X	X
<1000	0.72‡	0.38±0.08	<0.001	0.52	−0.01±0.24	0.96
1000–10000	0.88‡	0.53±0.08	<0.001	0.78	0.24±0.25	0.33
10000–100000	0.79‡	0.45±0.08	<0.001	0.85	0.31±0.25	0.21
>100000	0.34‡	**Referent**	**-**	0.54	**Referent**	**-**
**Time from HIV Diagnosis to ART Start (years)**	X	X	X		−0.13±0.07	0.06
**AIDS Diagnosis (time updated)**						
No	0.78‡	**Referent**	**-**	0.58‡	**Referent**	**-**
Yes	0.08	−0.70±0.12	<0.001	0.46	−0.13±0.67	0.85
**Time since HIV Diagnosis (years)**	NA	0.17±0.02	<0.001	NA	0.28±0.09	0.002
**Time on ART (years, time updated)**	NA	−0.12±0.02	<0.001	X	X	X
**Time on class-specific ART (years, time updated)** ‡‡						
NRTIs‡‡	NA	−0.15±0.03	<0.001	NA	−0.23±0.12	0.05
PIs‡‡	NA	0.04±0.03	0.10	NA	0.03±0.09	0.71
NNRTIs‡‡	NA	0.05±0.03	0.06	NA	0.08±0.09	0.37

SE, standard error.

*Adjusted for all other variables as described in the Methods.

**p-value represents the comparison of subgroup vs. reference group for each level of the factor of interest. The estimated difference represents the difference in mean change in BMI between the subgroup and the referent. For example, in Model 1, the estimated difference of 2.03 for participants who were underweight at baseline indicates that the mean change in BMI was 2.03 kg/m^2^ higher when compared to participants of normal weight at baseline.

‡*P*<0.05, for comparison of mean change to zero.

‡‡The analyses for Model 1 were repeated, once examining ART of any type, and once examining antiretroviral classes individually. The analyses for Model 2 only included antiretroviral classes individually.

“X” indicates that the factor was not included in the model. “NA” indicates the summary was not applicable for this variable type.

Regarding antiretroviral medication use, longer cumulative time on ART was associated with smaller increases in weight gain (Table 3, Model 1). When considering cumulative time on individual drug classes (instead of cumulative time on any ART regimen), increased exposure to NRTIs was associated with smaller increases in weight gain (p<0.001; Table 3, Model 1, last three rows); NNRTIs and PIs were associated with greater increases in weight gain per year of exposure, but neither was significant in the multivariate model. As an example, a 30-year-old white male diagnosed in 1990 with seven years of follow-up who received both NRTIs and a PI for one year had an estimated BMI increase of 1.23 kg/m^2^ during follow-up. A person with the same characteristics with five years of NRTI and one year of PI exposure had an estimated increase in BMI of 0.63 kg/m^2^. Specific NRTIs, including the thymidine analogs (zidovudine and stavudine) as well as didanosine and zalcitabine, were associated with less weight gain, whereas the other NRTIs were not significantly associated with weight change (data not shown).

We repeated the adjusted multivariate model for patients diagnosed in the HAART era (n = 630). Results were similar, except that an AIDS event was not associated with less weight gain, likely due to the small number of events in the HAART era (data not shown). We also examined factors associated with changes in BMI before use of antiretroviral agents among patients diagnosed in the HAART era and found higher time-updated CD4 count (estimate 0.25, p = 0.05), lower time-updated HIV-RNA level (HIV-RNA<1000 copies/mL vs. ≥100000 copies/mL, estimate 0.58, p = 0.01) and longer duration of HIV infection (estimate 0.26, p<0.001) remained associated with greater weight gain (data not shown). Finally, we examined participants diagnosed in the HAART era and after initiation of HAART (Table 3, Model 2); factors associated with more weight gain included longer duration of HIV infection, whereas factors associated with less weight gain included male gender, being obese, and early stage infection (all p<0.05). Increased exposure to NRTIs had a trend towards less weight gain. NNRTIs or PIs in the univariate and multivariate models were not significantly associated with weight gain. We repeated Models 1 and 2 separately by gender and found similar results.

## Discussion

Our study demonstrates a high prevalence of being overweight or obese among HIV-infected patients in the U.S. We found that 46% of persons in our HIV cohort were overweight or obese at the time of HIV diagnosis, a percentage that steadily increased from 28% in 1985–1990 to 53% in 1996–2004. Given that our participants were diagnosed early in the course of HIV infection due to military testing policies, these data may be a reflection of the growing obesity epidemic in the general population [1]. Since U.S. HIV testing guidelines have been expanded [15], our data provide important information about weight trends among persons diagnosed early in infection.

Our study also demonstrated that most HIV-infected patients are gaining weight during their infection, rather than experiencing weight loss or becoming underweight, which characterized the early epidemic. Among those diagnosed in the HAART era, nearly two-thirds of HIV-infected patients were overweight or obese at last visit. This percentage is similar to both the U.S. general and military populations [16], [17], suggesting that as HIV has become a chronic disease, HIV-infected patients' weights may be normalizing to the general population. Although encouraging in terms of the ability of antiretroviral therapy to reduce the occurrence of end-stage disease and wasting, HIV clinicians now need to be cognizant of weight excess among their patients.

From published data, the proportions of HIV-negative military members who were overweight or obese in 2005 were 60% and 12%, respectively [17]. Among our cohort of HIV-infected military beneficiaries, these proportions were 56% and 15% at the last study visit, respectively. These data suggest that prevalence of being overweight or obese among HIV-positive military beneficiaries is similar to that of HIV-negative persons within the military.

Although studies have suggested that wasting may remain common [3], [4], [18], more recent studies have found that obesity was more common than wasting [2], [10]. Disparate study results may be related to differences in timing of HIV diagnosis and treatment, socioeconomic status, and access to medical care. Of note, our population was diagnosed early in infection, had stable incomes, adequate food supplies, and free access to medical care. These data suggest that early diagnosis as supported by the recent CDC guidelines [15] and optimized medical care may be important factors in preventing AIDS-related wasting, which may, in turn, improve survival [19]–[21].

Several factors were associated with a greater increase in BMI in our study. Greater increase in weight gain in more recent years was likely related to the positive effects of antiretroviral therapy on preventing HIV-related complications, including wasting. Concurrent with this finding was that improved HIV status, as measured by the lack of AIDS diagnosis, lower HIV RNA levels, and high CD4 counts, was associated with increased weight gain similar to other studies [11], [22], [23]. An additional reason for weight gain may be the rising prevalence of weight excess in the general population [1].

Lower BMI at HIV diagnosis was also linked to more weight gain; although the exact nature of this finding is unclear, patients who initially had more advanced disease and lower weights may have become healthier and gained weight over time. Another possible explanation is that HIV-infected patients who perceive that they have a low BMI may gain more weight in an attempt to obscure their diagnosis [24]. Finally, duration of HIV was associated with weight gain; this observation is of particular interest, since long-term HIV infection, in the past, was associated with being underweight, rather than weight excess.

We found no association between ART and increased weight gain in the HAART era. In fact, antiretroviral use was associated with less weight gain. Specifically, NRTI use (zidovudine and the “D” drugs) was associated with less weight gain. This finding concurs with the lipoatrophic effects of some NRTI agents [25]. We found no significant relationships between NNRTIs or PIs and a greater change in BMI during the HAART era in our adjusted multivariate models. Our study did not collect information on weight distribution; as a result, we were unable to determine if various antiretroviral medications were related to differing patterns of lipodystrophy.

Our study has the advantage of examining longitudinal ART use in a clinical practice setting. Short-term studies have shown that ART initiation leads to weight gain, but this often tapers off or reverses over time [25]–[27]. Our population initiated HAART at a mean CD4 count of 384 cells/mm^3^; although HAART's effect on weight gain among end-stage patients may differ, our data provide important information among those initiating HAART by the recent treatment guidelines. Other studies on weight patterns have also shown no clear relationship between weight and HAART [2], [5], [10]. Together, these data suggest that ART may not play a direct role in causing excess weight and that the weight gain seen in the HAART era may be more related to improved health status rather than to direct ART effects.

The adverse health consequences of weight excess are well-described in the general population [28], [29], but limited data exist among HIV-infected patients. Our participants who were overweight or obese at last visit had a higher incidence of hypertension and hyperlipidemia. In the general population, excess weight contributes cardiovascular disease [29] and other medical conditions; the high rates of hyperlipidemia, hypertension, insulin resistance, and cardiovascular disease among HIV-infected patients [30]–[32] may also be partly due to excess weight. The increasing number of these medical complications may also be related to the use of certain antiretroviral medications, including protease inhibitors. Beyond the health consequences, these comorbidities impact healthcare costs; the complications associated with being overweight or obese accounts for nearly 10% of the total U.S. medical expenditures in the general population [33]. Applying these estimated costs to HIV-infected persons [33], if 46% and 16% of the 1.1 million HIV-infected patients in the U.S. are overweight or obese, respectively, this could result in an extra $250 million per year of healthcare costs among HIV-infected persons. Finally, since obesity contributes to lower life expectancies in the general population [28], [34], it is conceivable that it could also negatively impact survival among HIV-infected patients. These data suggest that weight management strategies are urgently needed components of HIV care which have the potential of reducing medical comorbidities, healthcare costs, and mortality among HIV-infected persons [35], [36].

Study limitations include the predominant male population evaluated; although the U.S. HIV population is predominantly male, our results cannot be generalized to women. We did, however, perform separate analyses by gender in our cohort. Since we evaluated a military cohort consisting of patients with early diagnosis, stable income, access to food, and free care, some of our results may not be generalizable to the overall HIV population and specifically to patients diagnosed late or with limited medical access. However, our study provides important data on weight trends among HIV-infected persons in the setting of optimized medical and social support. Data on physical activity, tobacco use, family history, or diet were not available; previous studies have shown that HIV-infected patients often ingest high fat diets, which likely affects rates of obesity [37]–[39]. Another limitation of our study is the lack of an HIV-negative control group for comparison. Furthermore, capture of medical conditions may have changed over time which may have limited our findings regarding these outcomes. Finally, although BMI is a well-recognized tool for weight assessment, it does not capture information on body habitus and may lack accuracy in certain ethnicities including African Americans whereby it may overestimate the prevalence of obesity [40]. Caliper measurements to determine percent body fat in various body sites would have been helpful in determining fat distributions and weight assessments in this study and are often conducted among military members; however, these data were not available in our study database. Since BMI does not depict changes in regional body fat distribution (i.e., central obesity) often seen among HIV-infected persons, future studies should capture information on body fat distribution.

In summary, HIV-infected persons in the U.S. are increasingly overweight and obese with rates of weight excess similar to the general population. Weight excess in the HIV-infected population is associated with adverse medical consequences, such as hypertension and dyslipidemia. Clinicians should be aware of these trends and consider instituting weight management programs as part of routine HIV care.
